# A Uterine Polypus Removed with the Aid of Ergot

**Published:** 1869-09-15

**Authors:** R. F. Henry

**Affiliations:** Princeville, Ill.


### *A Uterine Polypus Removed with the Aid of Ergot.*

BY R. F. HENRY, M.D., OF PRINCEVILLE, ILL.

Mrs. A———, aged about forty years, married to her second husband. Had never borne any children, but at the time above mentioned had some prominent symptoms of pregnancy. She had uterine hæmorrhage, and was supposed to be threatened with an abortion when I was consulted. An examination of her vagina revealed a dilatable os sincæ, and a tumor within the cavity of the uterus, very nearly the form and size of a fœtal head at six or seven months (judging from the part presenting and within the reach of the fingers). Not being satisfied, at this time, as to the nature of the case, I administered internal styptics, and succeeded in controlling the hæmorrhage for the present, and the patient got along very comfortably for several weeks; but the hæmorrhage returned several times during the summer, with varied severity, and finally, in December, 1857, a more violent attack than usual came on, which did not yield to the ordinary treatment. I then administered ergot in repeated doses, which not only controlled the hæmorrhage immediately, but in a few hours, forced the tumor through the os externum. It was then discovered that the tumor was attached to the fundus, had dragged it down with it, and thus partially inverted the uterus. The tumor was fibrinous, pear-shaped, with a short neck, and thin near the place where it originated. With the assistance of my friend, Dr. L. M. Andrews, we placed a ligature around the neck, and removed the tumor with a knife. The uterus immediately returned to its natural position. The ligature was withdrawn in a few days, and the patient recovered without any serious difficulty. The weight of the tumor was two and three-fourth ounces.

